# Results of Automated Retinal Image Analysis for Detection of Diabetic Retinopathy from the Nakuru Study, Kenya

**DOI:** 10.1371/journal.pone.0139148

**Published:** 2015-10-01

**Authors:** Morten B. Hansen, Michael D. Abràmoff, James C. Folk, Wanjiku Mathenge, Andrew Bastawrous, Tunde Peto

**Affiliations:** 1 NIHR Biomedical Research Centre at Moorfields Eye Hospital NHS Foundation Trust and UCL Institute of Ophtalmology, London, United Kingdom; 2 Research Unit of Ophthalmology, University of Southern Denmark, Odense, Denmark; 3 Department of Ophthalmology and Visual Sciences, University of Iowa Hospital and Clinics, Iowa City, IA, 52242, United States of America; 4 Rwanda International Institute of Ophthalmology, P.O. Box 312, Kigali, Rwanda; 5 International Centre for Eye Health, Department of Clinical Research, Faculty of Infectious and Tropical Diseases, London School of Hygiene and Tropical Medicine (LSHTM), London, United Kingdom; Justus-Liebig-University Giessen, GERMANY

## Abstract

**Objective:**

Digital retinal imaging is an established method of screening for diabetic retinopathy (DR). It has been established that currently about 1% of the world’s blind or visually impaired is due to DR. However, the increasing prevalence of diabetes mellitus and DR is creating an increased workload on those with expertise in grading retinal images. Safe and reliable automated analysis of retinal images may support screening services worldwide. This study aimed to compare the Iowa Detection Program (IDP) ability to detect diabetic eye diseases (DED) to human grading carried out at Moorfields Reading Centre on the population of Nakuru Study from Kenya.

**Participants:**

Retinal images were taken from participants of the Nakuru Eye Disease Study in Kenya in 2007/08 (n = 4,381 participants [NW6 Topcon Digital Retinal Camera]).

**Methods:**

First, human grading was performed for the presence or absence of DR, and for those with DR this was sub-divided in to referable or non-referable DR. The automated IDP software was deployed to identify those with DR and also to categorize the severity of DR.

**Main Outcome Measures:**

The primary outcomes were sensitivity, specificity, and positive and negative predictive value of IDP versus the human grader as reference standard.

**Results:**

Altogether 3,460 participants were included. 113 had DED, giving a prevalence of 3.3% (95% CI, 2.7–3.9%). Sensitivity of the IDP to detect DED as by the human grading was 91.0% (95% CI, 88.0–93.4%). The IDP ability to detect DED gave an AUC of 0.878 (95% CI 0.850–0.905). It showed a negative predictive value of 98%. The IDP missed no vision threatening retinopathy in any patients and none of the false negative cases met criteria for treatment.

**Conclusions:**

In this epidemiological sample, the IDP’s grading was comparable to that of human graders’. It therefore might be feasible to consider inclusion into usual epidemiological grading.

## Introduction

Regular screening for diabetic retinopathy (DR) for at risk populations has been shown to be an effective public health intervention for reducing the burden of disease in people living with diabetes mellitus (DM), as DR is still one of the leading causes of visual impairment in industrialised countries. [1, 2] The latest global estimates show that there are 285 million people with visual impairment, of whom 39 million are blind, with DR contributing to 1% of both to blindness and visual impairment. [3] The low-income countries in Asia and Africa have both the highest prevalence of DM and the highest expected rise in diseased population[4], but these resource-poor communities lack infrastructure to implement organised DR screening programs (DRSP) where regular eye exams [5, 6] can minimise the risk of visual loss. For example with only 2.7 ophthalmologists per million people in Sub-Saharan Africa, recommending the addition of DRSP in the format it is currently delivered in more developed countries is extremely challenging. [7, 8] Even in developed countries, such as the US, less than 60% of the 23 million people with DM had an eye examination in 2010. [7, 9] On the contrary, in the UK, where the DRSP is publicly funded, nearly 80% of DM patients are screened annually, [5], and as a result of this, DR is no longer the leading cause of blindness in the working age-group. [6]

Potentially, algorithms performing automated analysis of retinal images may address the need for more affordable DRSP in certain settings. The Iowa Detection Program (IDP) was originally designed to meet the rising demand for DRSP [10, 11]. IDP evaluates digital retinal images in an automated fashion for the presence of moderate or more severe DR as well as diabetic macular edema (DME) [10]. The IDP has been validated in independent cohorts of people with DM, using internationally recognized DR grading standards [9, 12] and was reported to have diagnostic accuracy comparable to that of fellowship-trained retinal specialists’. [10, 11]However, the performance of IDP is yet to be evaluated on populations from Sub-Saharan Africa, especially in the context of a population-based study where majority of the subjects are without DM.

The aim of this study is to determine the sensitivity, specificity, positive and negative predictive value of IDP in detecting Diabetic Eye Disease (DED) and non-DED in the population based Nakuru Study from Kenya. Human grading for DR used in this study was that carried out in the Moorfields Reading Centre, London, UK.

## Methods

### Subject recruitment and imaging

Images and clinical data of participants of the Nakuru Eye Study, Kenya, were used for the automated image analysis via the IDP software. The Nakuru Eye Study started in 2007/08 where a total of 4,381 participants underwent complete ophthalmic examination, and in 3460 subjects, fundus imaging was performed and subsequently graded for DR. [13]

In summary, for fundus photography participants were dilated with Mydriacyl drops (Alcon®) and retinal photographs were obtained using a Topcon® NW6S Non-Mydriatic Retinal Camera. Two fields were imaged, one centred on the disc, one on the fovea. [13] All fundus photographs were graded by human graders at Moorfields Eye Hospitals Reading Centre, UK. [14] The grading protocol was as follows:

### The grading protocol and ICDR reference standard

The human graders were masked to the patient’s status to having or not having DM. Table 1 Shows details of the grading scheme for human graders, in short, no retinopathy, mild, moderate and severe non-proliferative DR (NPDR) and proliferative DR (PDR) characteristics were determined by identifying individual lesions in the retina and the macular region. These lesions were microaneurysm, haemorrhages, exudates, characteristics of clinically significant macular edema (CSME) without being able to determine thickness due to lack of stereo imaging, cotton wool spots (CWS), intraretinal microvascular abnormalities (IRMA) and new vessels on the disc or elsewhere. In addition vascular abnormalities resembling new vessels characteristic of DR were graded as referable retinopathy as well.

**Table 1:** Grading protocol ICDR and ETDRS Severity Levels.

Measure	Score	Observable Findings
**ICDR severity level**		
No apparent retinopathy	0	No abnormalities (Level 10 ETDRS)
Mild non-proliferative diabetic retinopathy	1	Microaneurysm(s) only (Level 20 ETDRS)
Moderate non-proliferative diabetic retinopathy	2	More than just microaneurysm(s) but less than severe non-proliferative diabetic retinopathy (Level 35, 43, 47 ETDRS)
Severe non-proliferative diabetic retinopathy	3	Any of the following: > 20 intra-retinal haemorrhages in each of 4 quadrants, definite venous beading in ≥2 quadrants, prominent intra-retinal microvascular abnormalities in ≥1 quadrant, or no signs of proliferative retinopathy. (Level 53 ETDRS: 4-2-1 rule)
Proliferative diabetic retinopathy	4	One or more of the following: neovascularization and/or vitreous or preretinal haemorrhages. (Levels 61, 65, 71, 75, 81, 85 ETDRS)
**Macular oedema severity level**		
No macular oedema	0	No exudates and no apparent thickening within 1 disc diameter from fovea
Macular oedema	1	Exudates or apparent thickening within 1 disc diameter from fovea

Abbreviations: ETDRS, Early Treatment Diabetic Retinopathy study; ICDR, International Clinical Diabetic Retinopathy

### Iowa Diabetic Retinopathy (IDP) detection software

The details of the Iowa Detection Program (IDP) algorithm have been published in previous papers. [9–11] IDP examines and analyses every pixel in an image to detect microaneurysms, haemorrhages, exudates and cotton wool spots and has the ability to detect retinal neovascularization. It combines image quality with its detection of lesions and makes a numerical output between 0 and 1, called the dr-index. The closer the number to 1 the more likely that the patient has DED, or that the image was ungradable. Both of these categories (DED and ungradable) then is required to be regarded by a human grader. The dr-index is compared to the so-called set-point to give the final result of the analysis. The lower the set-point of the IDP, the higher the sensitivity, with increases the likelihood of false positives (overcalls), and in turn lowers specificity. Based on previous work[9, 11], 0.04 as a setpoint was found to be ideal to strike a balance between sensitivity and specificity as at this level a sensitivity of 91.6% was established. [11]

### Methods of comparing human grading to IDP output

In order to be able to compare human grading output to DED output of IDP, the human grading had to be re-categorized as following: no-DED includes none/mild DR with no macular oedema (denoted with 0); while DED includes levels of DR that are either currently sight threatening (severe NPDR and above) or that has a higher chance of developing into sight threatening disease within a year, such as moderate NPDR. In summary, the algorithm is set to detect moderate to severe non-proliferative DR (NPDR), proliferative DR (PDR), or Diabetic Macular Edema (DME), denoted as 1 for statistical purposes.

The IDP processed all available images. Once IDP results were locked using the predefined set-point the human grading results were re-coded to reflect the IDP’s outcome as above, so statistical analysis and like-for-like comparison could be made. Once the grading results were brought into a comparable outcome, we reviewed the results and statistical analysis took place.

As mentioned the IDP categorises results as DED (moderate NPDR or above) present or absent. Ungradeable images also obtain the value of 1, meaning DED due to the fact that these images require human grading intervention, therefore for fair comparison, the image sets were also compared including the ungradables.

### Primary outcome measure

The primary outcomes were sensitivity, specificity, positive and negative predictive value of IDP versus the human grading.

### Statistical analysis

Statistical analysis was performed using SPSS, version 22.0.0.0 (IBM Corporation, Worldwide). Sensitivity, specificity, negative predictive value (NPV), positive predictive value (PPV) of IDP compared to the reference standard of human grading were calculated.

Two methods were used to calculate these values. First we used the IDP’s output of DED and ungradeable/missing images were classified as a positive outcome and compared to the same setting for the human grading. Second method we used only results from gradable images to be analysed, both by the IDP and by the human grading for comparison.

### Ethics approval

The Nakuru Eye study was granted ethical approval for their work granted by the London School of Hygiene & Tropical Medicine, the Kenya Medical Research Institute (KEMRI) Ethical Committee and African Medical and Research Foundation (AMREF). Informed consent was obtained from the participating subjects. [13]

## Results

All available baseline images’ from the Nakuru study (6788 fundus photos of 3,460 participants) were graded both by human graders and IDP. Altogether, 132 eyes could not be photographed at all, and these were therefore not available for grading and subsequent statistical analysis.

Of the 3,460 participants, the human graders determined that 113 subjects had DED, giving a prevalence of 3.3% (95% CI, 2.7–3.9%). There were 56 cases with moderate NPDR, 20 with severe NPDR and 37 cases with PDR. In the Nakuru study altogether 6.5% of patients had diabetes, giving a 50.8% prevalence of DR in this cohort. [14]

Of the 3,460 participants, there were 91 true positive DED, 900 false positive DED, 2093 true negative DED and 22 false negative DED results. In 334 cases both the IDP and human graded the image to be ungradable and therefor as a positive outcome. In 20 cases the human graded the images as ungradable were the IDP analysed the images as sufficient for grading but with no-DED.

First we analysed the total agreement between IDP and human grading including ungradable results. Sensitivity of the IDP to the RC was 91.0% (95% CI, 88.0–93.4%), and specificity was 69.9% (95% CI, 68.3–71.6%). The PPV was 32.1% (95% CI, 29.6–34.7%), whereas the NPV was 98.0% (95% CI, 97.4–98.6%). (Table 2)

**Table 2:** Results—Comparison of the grading of DED from the IDP and the Reading Centre.

Reading Centre					
		DED	No-DED	Ungradable	
	DED	91	900	0	991
**IDP**	No-DED	22	2093	20	2135
	Ungradable	0	0	334	334
		113	2993	354	3460

**Fig 1:**
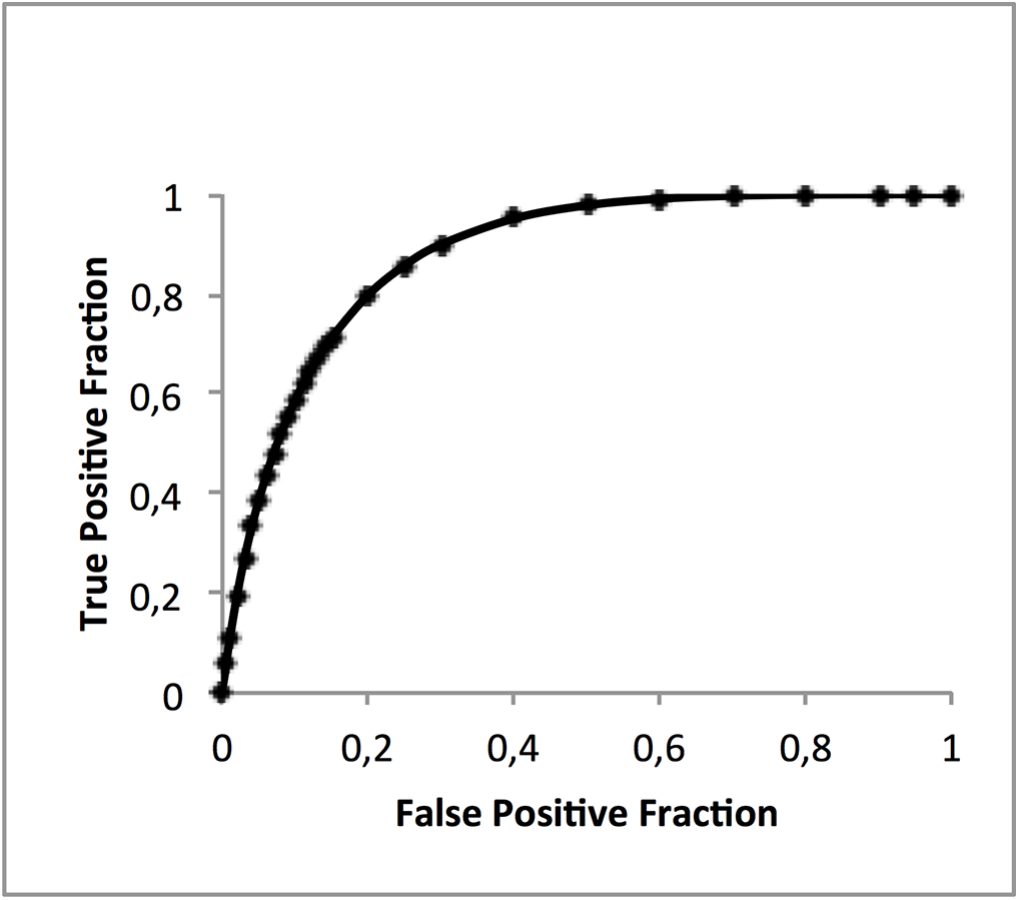
–The ROC.

The IDP ability to detect DED gave in Fig 1 an AUC of 0.878 (95% CI 0.850–0.905) using maximum likelihood estimation. [15]

Then we analysed the results without the ungradable images. We used the 91 true positive, 900 false positive, 2093 true negative and 22 false negative. The sensitivity was 80.5% (95% CI, 72.0–87.4%) and the specificity was 69.9% (95% CI, 68.3–71.6%). The PPV was 9.2% (95% CI, 7.5–11.2%) whereas the NPV was 99.0% (95% CI, 98.4–99.4%). (Table 2)

**Fig 2:**
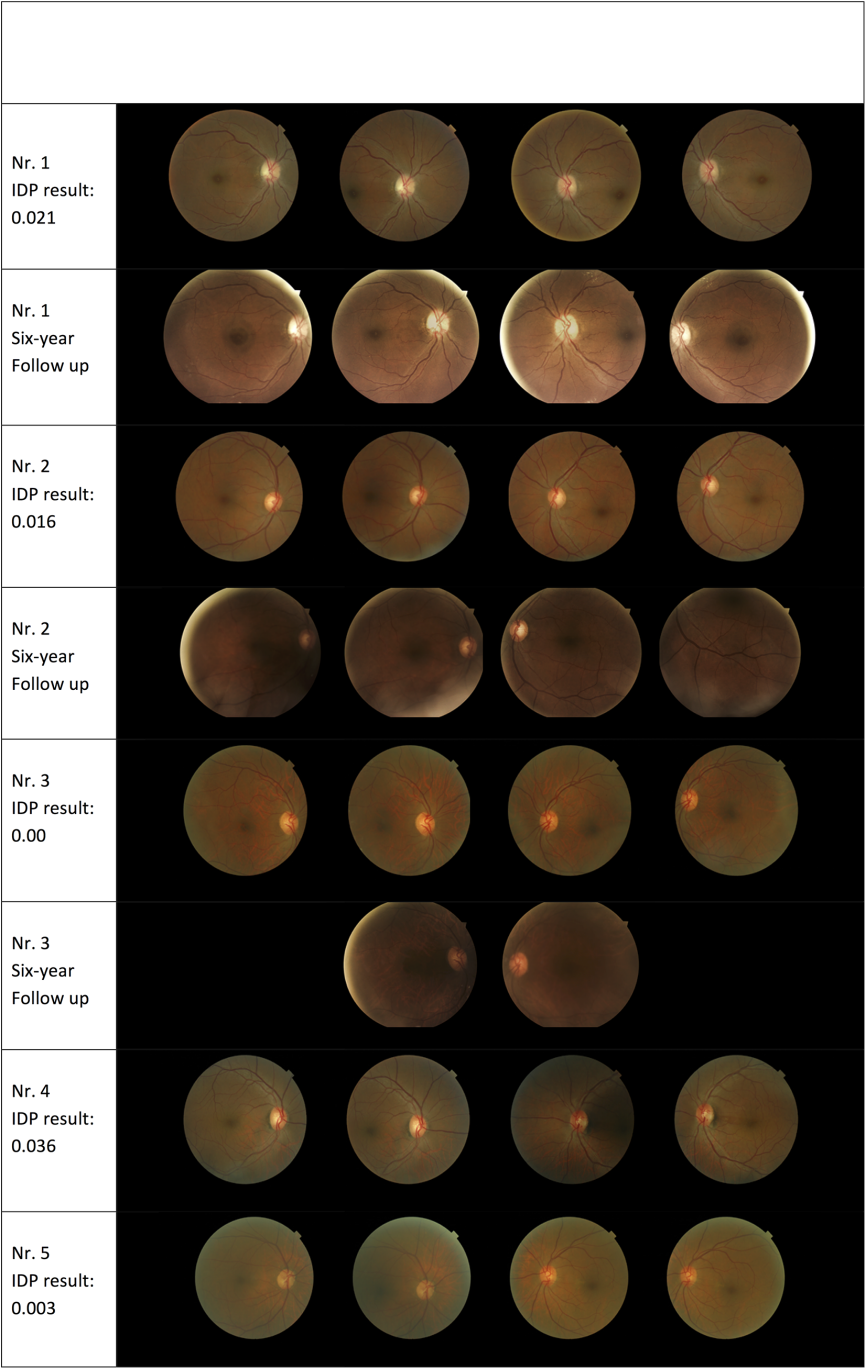
–Five false negative cases.

Of the 22 false negative cases (42 originally but 20 cases due to ungradable images), the human graders classified five cases as PDR, two as severe NPDR, and 15 as moderate NPDR. A retinal specialist, TP, reviewed these images a second time. The five cases read as PDR are shown in Fig 2. Review by the retinal specialist found none to have DR, but all were vascular abnormalities on the optic disc resembling PDR. Of the 2 cases graded as severe NPDR, both were confirmed to have haemorrhages and vascular abnormalities as IRMA, while of the 15 cases of moderate NPDR per the RC, three cases were re-adjudicated as mild DR while 12 remained at moderate level. The 12 cases of moderate NPDR were confirmed to be accurate with five showing only haemorrhages, three with IRMA and six cases showing a combination of small haemorrhages, IRMA and CWS.

Table 3 shows the results re-calculated once the adjudication results were taken into account. The modified sensitivity was 92.6% (95% CI, 89.7–94.7%), specificity 70.0% (95% CI, 68.3–71.6%), PPV 32.1% (95% CI, 29.6–34.7%) and NPV was 98.4% (95% CI, 97.8–98.9%).

**Table 3:** Results—Comparison of the grading of DED from the IDP and the Reading Centre–After review grading by retinal specialist.

		Reading Centre			
		DED	No-DED	Ungradable	
	DED	91	900	0	991
**IDP**	No-DED	14	2101	20	2135
	Ungradable	0	0	334	334
		105	3001	354	3460

Again after adjudication we performed analysis without the ungradable participants. This analysis resulted in 91 true positive, 1234 false positive, 2121 true negative and 14 false negative. This meaning a sensitivity of 86.7% (95% CI, 78.6–92.5%), and the specificity was 70.0% (95% CI, 68.3–71.7%). The PPV was 9.2% (95% CI, 7.5–11.2%) and the NPV was 99.3% (95% CI, 98.9–99.6%).

## Discussion

Overall, using automated image analysis software designed to detect sight threatening DR or ungradable images on a population based sample at Nakuru district of Kenya, sensitivity of 91% and specificity of 69.9% were achieved with excellent NPV value of over 99%. No vision threatening DR was missed in any patients. None of the false negative cases were found to have vision-threatening DED by the human graders or met criteria for treatment. Delayed treatment could have resulted from these missed cases; it would have been unlikely to lead to visual loss and high-risk PDR within the recommended year of follow-up. [16]

This is the first time IDP has been used on an epidemiologic population sample from Africa, i.e. not a set of people previously diagnosed with diabetes. With dark fundi in Sub-Saharan populations there has been concern that this would lead to underestimation of DED by the algorithms because lesions might have lower contrast with the retinal background. Our analysis did not show any such underestimation, and results were comparable to earlier studies in predominantly white / Caucasian populations. [9] The IDP performed well and it showed good results in differentiating participants into having no DED and having DED as a primary population screening tool and achieved this within the similar timeframe of one minute per eye as previously documented. [9] With DM becoming a major public health issue worldwide and a 69% rise estimated in the number of adults with DM in low-income countries [17], the need for cost-effective DRSP is essential. This opens up the opportunity for population based screening programs all over the world speed up the disease detection process from image acquisition to diagnosis and potentially reporting back to the outcome for timely treatment to be given. By reliably identifying patients who are in need of further image analysis by human graders and who potentially require treatment for DR conserves resources to those truly needing it. This could be of value in low-income countries where ophthalmic image grading is regularly carried out by the small number of ophthalmologists who are already overstretched coping with the demand. [18] Software that automatically detects sight threatening DR and ungradeable images in at risk patients could be part of the solution to that. As if IDP can cut down on the number of images to be seen by a human grading (in many cases an ophthalmologist) by over 60% as it did in this study, it would reduce the burden on health systems.

Even though the IDP today delivers good results there are ways to improve. The IDP focuses on detecting microaneurysms, haemorrhages, and exudates. Better detection of subtle changes such as IRMA or beading and CWS may improve on the detection level between DED and non-DED. This should be a focus point for future improvements to the algorithm.

At the moment the software only focuses on DR and no other eye diseases such as Age-Related Macular Degeneration (AMD) and glaucoma are covered and no other major blinding diseases are classified as abnormal by the software. For a complete clinical evaluation, the patients’ retinal images still need to be seen by trained graders or ophthalmologist. Combining software that screens for both DR, AMD and glaucoma would be the ideal solution for automated image analysis to lower the burden on healthcare worldwide. This should be an aim for the future.

In conclusion, IDP performed well in detecting and differentiating between participants with or without sight threatening DED and identified those with too poor quality images appropriately. The IDP showed its ability to cut down on the images needed to be seen by a grader by over 60%, provided only the device’s positive DED outputs were to be reviewed by a human grader. Further improvements are required to enable automated image analysis for all common diseases so large scale epidemiological studies, such as the Nakuru Eye Study, can be safely graded with minimal human intervention.

## References

[1] Ding J, Wong TY. Current epidemiology of diabetic retinopathy and diabetic macular edema. Current diabetes reports. 2012;12(4):346–54. 10.1007/s11892-012-0283-6 . 22585044

[2] Cheung N, Mitchell P, Wong TY. Diabetic retinopathy. Lancet. 2010;376(9735):124–36. 10.1016/S0140-6736(09)62124-3 . 20580421

[3] Pascolini D, Mariotti SP. Global estimates of visual impairment: 2010. The British journal of ophthalmology. 2012;96(5):614–8. 10.1136/bjophthalmol-2011-300539 . 22133988

[4] Stevens GA, White RA, Flaxman SR, Price H, Jonas JB, Keeffe J, et al Global prevalence of vision impairment and blindness: magnitude and temporal trends, 1990–2010. Ophthalmology. 2013;120(12):2377–84. 10.1016/j.ophtha.2013.05.025 . 23850093

[5] NHS Diabetic Eye Screening Programme [Access Date; 24 February 2015]. Available: http://diabeticeye.screening.nhs.uk.

[6] Liew G, Michaelides M, Bunce C. A comparison of the causes of blindness certifications in England and Wales in working age adults (16–64 years), 1999–2000 with 2009–2010. BMJ Open. 2014;4(2):e004015 10.1136/bmjopen-2013-004015 24525390 PMC3927710

[7] Resnikoff S, Felch W, Gauthier TM, Spivey B. The number of ophthalmologists in practice and training worldwide: a growing gap despite more than 200,000 practitioners. The British journal of ophthalmology. 2012;96(6):783–7. 10.1136/bjophthalmol-2011-301378 . 22452836

[8] Palmer JJ, Chinanayi F, Gilbert A, Pillay D, Fox S, Jaggernath J, et al Mapping human resources for eye health in 21 countries of sub-Saharan Africa: current progress towards VISION 2020. Human resources for health. 2014;12:44 10.1186/1478-4491-12-44 . 25128163 PMC4237800

[9] Abramoff MD, Folk JC, Han DP, Walker JD, Williams DF, Russell SR, et al Automated analysis of retinal images for detection of referable diabetic retinopathy. JAMA ophthalmology. 2013;131(3):351–7. 10.1001/jamaophthalmol.2013.1743 . 23494039

[10] Abramoff MD, Niemeijer M, Suttorp-Schulten MS, Viergever MA, Russell SR, van Ginneken B. Evaluation of a system for automatic detection of diabetic retinopathy from color fundus photographs in a large population of patients with diabetes. Diabetes care. 2008;31(2):193–8. 10.2337/dc07-1312 18024852 PMC2494619

[11] Abramoff MD, Reinhardt JM, Russell SR, Folk JC, Mahajan VB, Niemeijer M, et al Automated early detection of diabetic retinopathy. Ophthalmology. 2010;117(6):1147–54. 10.1016/j.ophtha.2010.03.046 20399502 PMC2881172

[12] Wilkinson CP, Ferris FL 3rd, Klein RE, Lee PP, Agardh CD, Davis M, et al Proposed international clinical diabetic retinopathy and diabetic macular edema disease severity scales. Ophthalmology. 2003;110(9):1677–82. 10.1016/S0161-6420(03)00475-5 . 13129861

[13] Bastawrous A, Mathenge W, Peto T, Weiss HA, Rono H, Foster A, et al The Nakuru eye disease cohort study: methodology & rationale. BMC ophthalmology. 2014;14:60 10.1186/1471-2415-14-60 24886366 PMC4024270

[14] Mathenge W, Bastawrous A, Peto T, Leung I, Yorston D, Foster A, et al Prevalence and correlates of diabetic retinopathy in a population-based survey of older people in Nakuru, Kenya. Ophthalmic epidemiology. 2014;21(3):169–77. 10.3109/09286586.2014.903982 . 24758280

[15] Eng J. ROC analysis: web-based calculator for ROC curves Baltimore: Johns Hopkins University [updated 2014 March 19]. Available: http://www.jrocfit.org [7 April 2015].

[16] Fundus photographic risk factors for progression of diabetic retinopathy. ETDRS report number 12. Early Treatment Diabetic Retinopathy Study Research Group. Ophthalmology. 1991;98(5 Suppl):823–33. . 2062515

[17] Shaw JE, Sicree RA, Zimmet PZ. Global estimates of the prevalence of diabetes for 2010 and 2030. Diabetes research and clinical practice. 2010;87(1):4–14. 10.1016/j.diabres.2009.10.007 . 19896746

[18] Bastawrous A, Hennig BD. The global inverse care law: a distorted map of blindness. The British journal of ophthalmology. 2012;96(10):1357–8. 10.1136/bjophthalmol-2012-302088 22740107 PMC3457914

